# Prevalence and Reporting of Needle Stick Injuries: A Survey of Surgery Team Members in Kermanshah University of Medical Sciences in 2012

**DOI:** 10.5539/gjhs.v8n3p245

**Published:** 2015-08-18

**Authors:** Alireza Khatony, Alireza Abdi, Faranak Jafari, Kamran Vafaei

**Affiliations:** 1 Nursing and Midwifery School, Kermanshah University of Medical Sciences, Kermanshah, Iran

**Keywords:** surgeon, injury, needle stick

## Abstract

**Background:**

Surgeons are one of the groups, most highly exposed to the risk of needle stick injuries at work. The present study aims to determine the prevalence and reporting of needle stick injuries during the first 6 months of 2012, in faculty surgeons affiliated to the Kermanshah University of Medical Sciences.

**Methods:**

In a cross-sectional descriptive-analytical survey, 29 surgeons were studied based on the census method. A reliable and valid questionnaire was used as a research instrument to collect the data. Data was analyzed using SPSS v.16 and based on descriptive and inferential statistics.

**Results:**

Among 29 recruited surgeons, 5 (17.2%) had needle stick injuries during the 6 months, only one of whom had followed the established guidelines about reporting and following treatment. The most common instrument causing injury was the suture needle (60%). Significant differences were found in both groups of the injured and non-injured in term of gender (X2=5.612, P=0.003), and number of patients (Z=2.40, P=0.016) and daily working hours (Z=2.85, P=0.04).

**Conclusions:**

In relation to the relatively high prevalence of needle stick injuries among the surgeons and their lack of reporting, it is suggested that the Safety Guidelines in the operating room are carefully observed. Moreover, safer and lower risk surgical Instruments should be used.

## 1. Background

Needle stick injury is defined as a penetrating wound typically induced by a needle point or other sharp instrument or object which could be infected with another person's secretions (Galougahi, 2010). Approximately, 600, 000 to 800,000 cases of needle stick injuries happen in the USA annually (Rapparini, 2006). Findings of a

study in China (2011) indicate that each health care personnel experience at least 4 times needle stick injuries during their employment period (Shi et al., 2011). Despite thorough precautions needle stick injuries cannot be avoided. These injuries can lead to transmission of blood-borne viral infections such as AIDS, hepatitis B (HBV) and hepatitis C (HCV) to health care employees, patients and their family members. As a result, 6-30% of HBV transmissions, 5-10% of HCV infections and 0.03% AIDS transmission are due to needle stick injuries (Askarian & Malekmakan, 2006; Thomas & Murray, 2009). Veeken et al. (1991) and Rapparini (2006) stated that needle stick injuries are the most common cause of AIDS infection in health care workers (Rapparini, 2006; Veeken et al., 1991). The prevalence of needle stick injuries differs according to working conditions, specialization and work environment. Surgeons are more vulnerable to needle stick injuries due to continuous exposure to patients’ secretions and blood (OÇÖConnor et al., 2011). Results of a study in Iran indicated that operating rooms where the staff have the highest exposure to sharp instruments such as needles, suture needles and surgical blades had the highest prevalence of needle stick injuries (Galougahi, 2010). In another study in England, 19% of all the needle stick injuries in a health center were related to the operating rooms(Thomas & Murray, 2009). Despite the high prevalence of needle stick injuries, evidence indicates that surgeons do not report their injuries or take follow up remedial action due to lack of time, lack of belief in infection transmission through needle stick injuries and deficiencies in infection control systems (Kelly, 2009).

Due to the importance of the issue and uncertainty of prevalence and reporting of needle stick injuries in surgeon specialists affiliated to Kermanshah University of Medical Sciences (KUMS), this study was conducted to explain the prevalence and reporting of needle stick injuries in surgeons of KUMS during the first six months of 2012.

## 2. Methods

This study is a cross-sectional descriptive-analytical survey conducted in 2012 in the educational hospitals of KUMS. The population includes all the faculty surgeons of KUMS (n=37), a list of whom was received from the university's Deputy Chancellor of Health. Twenty nine of them were studied based on census method and others did not participate in the study due to unwillingness or not being available. Inclusion criteria were attendance at work during the first six months of 2012 and willingness to complete the questionnaire.

The research instrument used to collect the data was a two-part questionnaire. The first part was related to personal and occupational information, including 7 questions about age, gender, specialization, work experience in the medical profession, work experience as a surgeon, the mean number of operations per day and daily working hours.

The second part of the questionnaire included 12 questions about needle stick injury experiences, the instruments causing the injuries, the type of surgery, surgery duration times, the probable cause of injury, actions taken, and the prevalence of vaccination against HBV. The questionnaire was taken from an England study on “the prevalence and reporting of needle stick injuries in surgeons” by Thomas et al. (Thomas & Murray, 2009). First, the questionnaire was translated into Persian and then into English. The two translations were scrutinized for discrepancies. Content validity was considered the indicator of questionnaire validity. Hence, the questionnaire was given to 12 faculties and modified based on their comments. For internal consistency, Cronbach alpha was calculated for the questionnaire as 0.78.

In order to collect the data, written permission was first obtained from the KUMS Vice Chancellor for Research and Technology for this project, then it was approved as no. 91049. A list of surgeons and their work place was received from the health deputy of KUMS. Next, the researcher collected data by visiting the operating rooms. The aim of the study was explained to the participants, their informed consent was obtained, and then they were asked to complete the questionnaire. All the participants were assured of the confidentiality and anonymity of their personal information, following which the completed questionnaires were gathered. The time required for completing the questionnaires was less than 10 minutes. The data was collected over a period of 3 months from September to November, 2012.

Data was entered into the 16th version of the Statistical Package for Social Sciences (SPSS v.16.0; SPSS Inc., Chicago, IL, USA) software and analyzed by descriptive (frequency percent, mean and standard deviation) and inferential statistics (chi-square and Mann–Whitney U test). The chi-square was used to determine the differences between two groups of the injured and non- injured in terms of gender and specialization variables. The Mann–Whitney U test was implemented to compare the participants’ mean work experience, operation experience, number of operations performed and daily working hours in both groups. In order to determine the normality of quantitative variables (age, operation experience, daily working hours and number of operations per day) the Shapiroo-Wilk test was used, of which the P value was less than 0.05. The significance level for all tests was 0.05.

## 3. Results

### 3.1 Participant Characteristics

Of the 37 surgeon faculty members of KUMS, 6 persons (16.2%) were unwilling to participate in the study and 2 individuals (5.4%) were also not available due to absence. Therefore data was analyzed on 29 participants (78.4 %). Of the 29 participants, 26 (89.7%) were males and 3 (10.3%) females. The mean and standard deviation (SD) of age was 49.96 ± 7.45 yrs. Most of the participants specialized in general surgery (8 members, 27.6%), orthopedic (5, 17.2%), nephrology (5, 17.2%), and neurosurgery (4, 13.8%), respectively. Two specialists each (6.9%) in ophthalmology, cardiovascular surgery and ENT (ear, nose and throat), and one urologist, were the other research participants.

### 3.2 Comparing Injured and Non-Injured Groups

Over the past 6 months, 5 persons (17.2%) were injured, 3 of whom (60%) were female and 2 were male. The chi-square test showed a significant difference between the genders in both the injured and non-injured groups (*P* = 0.003, X^2^ = 12.56, df = 1). However, there was no significant difference between the two age groups. Of the total injury cases, 4 (80%) happened in the morning and the others (20%) occurred during the evening shift. The chi-square test found no significant difference between the two groups in terms of their work shifts.

About 82% of the sample (n=24) had been vaccinated against hepatitis B. The mean and SD of surgical work experience of the participants was 14±6.25 yrs. than it was 12.6±2.5 and 13.91±6.8 yrs. In the injured and non-injured groups respectively. There was no significant difference between the two groups in terms of surgical work experience. The mean and SD of daily work was 11.31±2.8 hours per day, this rate was higher in the injured group (11.95±2.6 vs. 8.2±0.83 hours). The Mann-Whitney U test showed that the two groups (the injured and non-injured) differed significantly in terms of daily work hours (P= 0.04, Z=2.85). The mean and SD number of daily surgeries was 7.85±3.31 cases, which was higher in the injured group than the non-injured group (8.25±3.22 vs. 4.4±1.34). The two groups differed significantly according to the number of surgeries per day (P= 0.016; Z= 2.40). (Table 1)

**Table 1:** comparison of the demographic characteristics between injured and non-injured groups

groups variables		Injured (means± SD)	Non-injured (means± SD)	Total (means± SD)	Statistical values
Age (year)		45.80± 4.81	50.83± 7.68	49.96± 7.45	Z= 1.35
					P= 0.174
working experience in medical profession (year)		16.60± 2.07	19.37± 6.51	18.89± 6.05	Z= 1.39
					P=0.165
working experience as surgeon (year)		12.6 ± 2.5	13.91±6.8	14.0±6.25	Z= 0.694
					P= 0.487
Operations numbers per day		8.25± 3.22	4.4±1.34	7.85 ± 3.31	Z= 2.40
					*P= 0.016
daily working hours		11.95± 2.6	8.2 ±0.83	11.31 ± 2.8	Z=2.85
					*P= 0.04
sex	male	2 (7.7%)	24 (92.3%)	26 (100%)	x^2^ = 12.56
	female	0.0%	3 (100%)	3 (100%)	*p=0.003
Injection HB Vaccine	yes	5 (20.8%)	19 (79.2 %)	24 (100%)	x^2^ = 2.09
	no	0 (0.0%)	5 (100%)	5(100%)	p=0.553

* significant.

### 3.3 Measures After Injury

**Figure 1:**
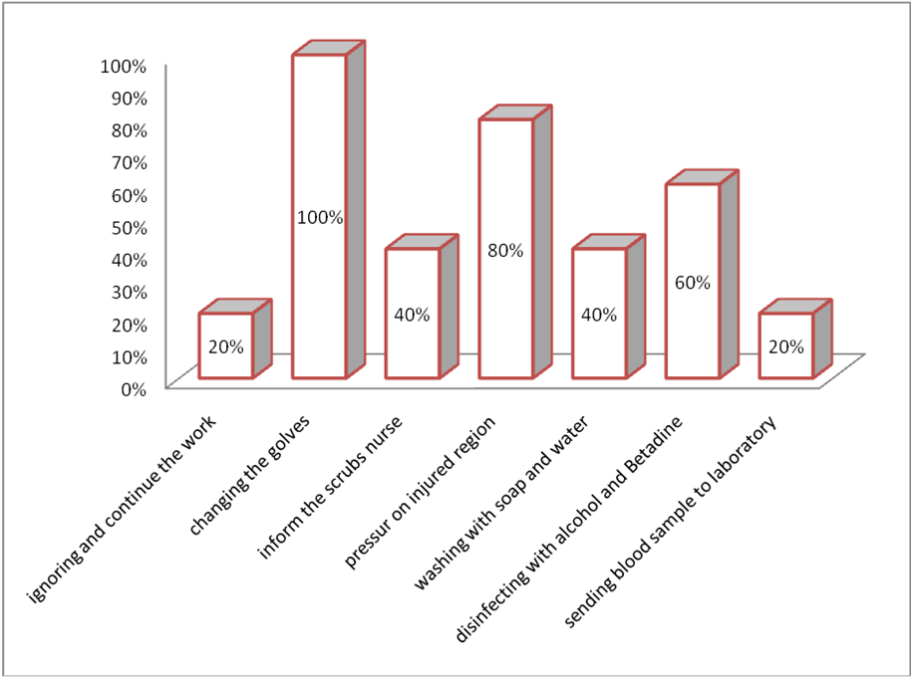
Frequency percentage of measures taken after needle stick injury of surgeons

The cause of 60% of the injuries was a suture needle, and the remaining 40%, 20% each were made by needles and scalpels. All of those who were injured (5 people) mentioned carelessness as the cause of injury. The most common measures taken after the injury by affected surgeons included changing their gloves (100%), pressure on the injured region (60%) and disinfecting with alcohol and Betadine (60%), respectively. Among those affected, only one participant was referred for blood tests in the laboratory (Figure 1).

## 4. Discussion

The purpose of this study was to evaluate the prevalence and reporting rate of needle stick injuries from sharp objects in surgeons. The results showed that the frequency of injuries was nearly high (17.2% over 6 month). In comparison, Thomas et al. (2009) reported the UK incidence of injury from sharp objects in surgeons as 44% over 6 months (Thomas & Murray, 2009). In another study conducted in China, at 93.09% during their working period, operating room staff and surgeons had the highest incidence of injury from sharp objects after the delivery ward staff, (Shi et al., 2011). In a German study compared with other medical hospital personnel, physicians had the highest rate of needle stick injury (Wicker et al., 2008). A study conducted in India, showed surgeon's assistants were more exposed to needle stick injuries than other health care workers (Rele, et al 2002).

Similar studies in Pakistan and England demonstrated working in operating rooms is the main factor increasing prevalence of needle stick injuries (Afridi et al., 2013; Gaballah et al., 2012). Results of these studies are consistent with findings of our research on needle stick injuries. In this regard, the researchers believed that the relatively high prevalence of needle stick injuries in surgeons is due to exposure to instruments such as scalpels, suture needles and sharp forceps, as well as failure to follow the instructions and standards for correct use of the instruments, not wearing protective equipment and not disposing of sharp objects after use, all play an important role in increasing the risk of injury in surgeons.

The most common cause of injury in our study was using the suture needle during surgery. In this regard, the study of Eftaei et al. (2009) in Nigeria also indicated that more than 86% of the injuries in surgeons have occurred during suturing (Efetie & Salami, 2009). In the study of Shi et al., the most common cause of injury was also syringes and needles, and suture needles were the cause of 17% of reported injuries (Shi et al., 2011). Lack of attention to the application of such sharp instruments as suture needles, which surgeons mostly deal with, and also using non-standard instruments could increase the risk of needle stick injuries. Hoffman and colleagues suggested that the main cause of injury was the improper use of equipment and sharp objects (Hofmann et al., 2002). Gurgia and De Weerd, (2009) have noted the use of safety equipment and taking safety precautions during procedures as important factors in reducing the injuries caused by sharp objects. In recent studies, it has been suggested that surgeons use new surgical techniques such as blunt suture needles and electrosurgical suspension apparatus to reduce the risk of needle stick injuries (Parantainen et al., 2011; Weber et al., 2000). Such measures as placing a tip along the needle forceps after using the suture needle, have been recommended to prevent accidental penetration of the skin (Gurgia & De Weerd, 2009). Providing standard and low risk instruments, as well as holding regular training courses on new techniques for their safe and low-risk operation, can also reduce the prevalence of needle stick injuries in surgeons.

The results of this study showed that the reporting rate in surgeons was low and follow-up treatment and tests after they received a needle stick injury was rare. Most considered replacing the gloves and disinfecting the injury site sufficient. The results of the study by Thomas and colleagues showed that only 9% of surgeons who suffered from needle stick injuries reported their injuries and more than 70% of them only took initial actions (Thomas & Murray, 2009). In a similar study in Nigeria, 9.2% of surgeons observed the protocols, and the reporting and follow-up interventions after experiencing injuries with sharp objects (Efetie & Salami, 2009). The results of the present study are consistent with the findings of these studies. Previous research on other medical personnel has recorded higher levels of reporting of needle stick injuries. For instance in a study by Khader et al. 23% of dentists injured by needles and sharp objects reported the incident (Khader et al., 2009). In another study conducted on health care workers in an Indian hospital, 68% of injuries from sharp objects were reported and follow-up action was taken (Jayanth et al., 2009). There are several reasons for non-reporting of needle stick injuries by surgeons. A UK study mentioned two causes, including low risk of contamination and time-consuming reporting processes (Wicker et al., 2008). Kennedy et al. also found surgeons’ fail to follow up and report injuries. Issues such as time-consuming processes, low transmission probability from sharp object injuries, reluctance to interrupt surgery and follow instrument use instructions, and the low efficacy of treatment and prevention of post-injury were also recorded by Kennedy et al. (2009). In the present study, the low number of reported injuries, in addition to the aforementioned reasons, were due to surgeons’ high workloads and difficulties following the post injury instruction processes.

In the current study, the rate of injury was significantly higher in females than males and the surgeons who performed more surgeries per day and worked more hours daily had higher injuries. In terms of experience, age and shift work, there was no significant difference between the injured and the non-injured groups. The research of Galogahi et al. in Tehran, showed that age, gender and work experience were not related to the frequency of injury from sharp objects incurred by medical staff (Galougahi, 2010). But Khader et al. stated that with the increasing the age of surgeons and the higher numbers of patients, the rate of injuries had increased significantly (Khader et al., 2009). In another study, the surgeons who had less experience were more susceptible to injury from sharp objects (Jayanth et al., 2009), and in a study by Kummar et al., the intensity of their daily work was mentioned as a predisposing factor to needle stick injury in medical staff (Kumar et al., 2011). This is in line with the current research findings. The excessive workloads and long working hours may lead to fatigue and poor concentration in surgeons, and thus increase the chance of injury from sharp objects.

## 5. Conclusions

The results indicate a relatively high prevalence of injuries from sharp objects and low levels of reporting and follow-up. Regular workshops on new techniques for safe operation are suggested to reduce the exposure of surgeons to injuries by sharp objects. Due to the small sample size of this study, it is difficult to determine the effect and relationship of gender, work experience, and the workload of surgeons on the frequency of needle stick injuries. Hence, more studies are necessary in this field. It is recommended that the factors associated with the follow-up and reporting of needle stick injuries in surgeons be assessed with a larger sample size.

## References

[ref1] Afridi A. A. K, Kumar A, Sayani R (2013). Needle stick injuries-risk and preventive factors: A study among health care workers in tertiary care hospitals in Pakistan. Global journal of health science.

[ref2] Askarian M, Malekmakan L (2006). The prevalence of needle stick injuries in medical, dental, nursing and midwifery students at the university teaching hospitals of Shiraz, Iran. Indian Journal of Medical Sciences.

[ref3] Efetie E. R, Salami H. A (2009). Prevalence of, and attitude towards, needle-stick injuries by Nigerian gynaecological surgeons. Nigerian journal of clinical practice.

[ref4] Gaballah K, Warbuton D, Sihmbly K, Renton T (2012). Needle stick injuries among dental students: Risk factors and recommendations for prevention. Libyan Journal of Medicine.

[ref5] Galougahi M. H. K (2010). Evaluation of needle stick injuries among nurses of Khanevadeh Hospital in Tehran. Iranian journal of nursing and midwifery research.

[ref6] Gurgia L, De Weerd L (2009). Technical Notes and Tips: Needle Locking Tip that Reduces Needle Stick Injuries. Annals of the royal college of surgeons of England.

[ref7] Hofmann F, Kralj N, Beie M (2002). [Needle stick injuries in health care-frequency, causes und preventive strategies]. Gesundheitswesen (Bundesverband der Arzte des Offentlichen Gesundheitsdienstes (Germany)).

[ref8] Jayanth S. T, Kirupakaran H, Brahmadathan K. N, Gnanaraj L, Kang G (2009). Needle stick injuries in a tertiary care hospital. Indian journal of medical microbiology.

[ref9] Kelly S (2009). Needle-stick reporting among surgeons. Annals of the royal college of surgeons of England.

[ref10] Kennedy R, Kelly S, Gonsalves S, Mc Cann P. A (2009). Barriers to the reporting and management of needlestick injuries among surgeons. Irish journal of medical science.

[ref11] Khader Y, Burgan S, Amarin Z (2009). Self-reported needle-stick injuries among dentists in north Jordan. Eastern Mediterranean Health Journal.

[ref12] Kumar N, Sharma P, Jain S (2011). Needle stick injuries during fine needle aspiration procedure: Frequency, causes and knowledge, attitude and practices of cytopathologists. Journal of cytology/Indian Academy of Cytologists.

[ref13] OGÇÖConnor M. B, Hannon M. J, Cagney D, Harrington U, OGÇÖBrien F, Hardiman N, OGÇÖConnor C (2011). A study of needle stick injuries among non-consultant hospital doctors in Ireland. Irish journal of medical science.

[ref14] Parantainen A, Verbeek J. H, Lavoie M. C, Pahwa M (2012). Blunt versus sharp suture needles for preventing percutaneous exposure incidents in surgical staff. Archivos de prevención de riesgos laborales.

[ref15] Rapparini C (2006). Occupational HIV infection among health care workers exposed to blood and body fluids in Brazil. American journal of infection control.

[ref16] Rele M, Mathur M, Turbadkar D (2002). Risk of needle stick injuries in health care workers-A report. Indian journal of medical microbiology.

[ref17] Shi C. L, Zhang M, Xie C (2011). [Study on status of needle-stick and other sharps injuries among healthcare workers in a general hospital]. Zhonghua lao dong wei sheng zhi ye bing za zhi=Zhonghua laodong weisheng zhiyebing zazhi=Chinese journal of industrial hygiene and occupational diseases.

[ref18] Thomas W. J. C, Murray J. R. D (2009). The incidence and reporting rates of needle-stick injury amongst UK surgeons. Annals of the royal college of surgeons of England.

[ref19] Veeken H, Verbeek J, Houweling H, Cobelens F (1991). Occupational HIV infection and health care workers in the tropics. Tropical doctor.

[ref20] Weber P. J, Moody B. R, Foster J. A (2000). Electrosurgical suspension apparatus. Dermatologic surgery.

[ref21] Wicker S, Jung J, Allwinn R, Gottschalk R, Rabenau H. F (2008). Prevalence and prevention of needlestick injuries among health care workers in a German university hospital. International archives of occupational and environmental health.

